# Pharmacological rescue of follicle-stimulating hormone receptor mutants. *In vitro* and *in silico* studies

**DOI:** 10.1210/endocr/bqag045

**Published:** 2026-04-16

**Authors:** Teresa Zariñán, Eduardo Jardón-Valadez, Rubén Gutiérrez-Sagal, Ernesto Ulloa-Pérez, Selvaraj Nataraja, Henry N Yu, Alfredo Ulloa-Aguirre

**Affiliations:** Red de Apoyo a la Investigación (RAI), National University of Mexico, Mexico City 04510, Mexico; Departamento de Recursos de la Tierra, UAM-Unidad Lerma, Edo. de México 52005, Mexico; Red de Apoyo a la Investigación (RAI), National University of Mexico, Mexico City 04510, Mexico; Department of Biostatistics, Epidemiology and Informatics, Perelman School of Medicine, University of Pennsylvania, Philadelphia, PA 19104, USA; Cancer Biology, Astellas Pharma, Cambridge, MA 02141, USA; CanWell Pharma Inc., Wellesley Hills, MA, 02481, USA; Red de Apoyo a la Investigación (RAI), National University of Mexico, Mexico City 04510, Mexico; RAI, Instituto Nacional de Ciencias Médicas y Nutrición Salvador Zubirán, Mexico City14000, Mexico

**Keywords:** follicle-stimulating hormone, follicle-stimulating hormone receptor, allosteric agonist, pharmacological chaperone, pharmacoperone, molecular dynamics

## Abstract

Mutations in the follicle-stimulating hormone receptor (FSHR) may result in impaired plasma membrane expression due to misfolding and intracellular retention of the receptor, leading to disease. Rescue of misfolded receptors may be achieved employing pharmacological chaperones (small molecules that specifically bind misfolded proteins, promoting their correct trafficking to their site of action). This study analyzed whether the small-molecule FSHR agonist CAN1405 rescued membrane expression and function of 13 mutant FSHRs leading to premature ovarian failure in women. FSHRs were expressed in HEK-293 cells, and membrane expression was assessed by immunoblotting before and after incubation with CAN1405. Three trafficking defective variants in the ectodomain of the FSHR (A189V, N191I, and D224V) and 3 others located in transmembrane domains (TMD) 3 and 4, and extracellular loop 2 (A462P, P504S, and P519T, respectively) failed to respond (or did it marginally) to CAN1405 by increasing their membrane expression. In contrast, in 7 variants located in the TMD2 (D408Y, A419T, and I423T), TMD6 (A575V, P587H, and F591S), and extracellular loop 3 (L597I), CAN1405 rescued membrane expression of the variants. Functional studies showed that after CAN1405 removal, rescued FSHRs responded to the orthosteric agonist in terms of cAMP-mediated signaling and ERK1/2 phosphorylation. Refined molecular dynamics simulations using the cryo-EM structure of the FSHR revealed key conformational changes and interactions within the TMDs provoked by CAN1405, highlighting potential allosteric binding sites critical for receptor activation. These findings offer a promising therapeutic strategy for treating mutation-provoked FSHR dysfunction and underscore the synergistic potential of computational biophysics in drug discovery.

Follicle-stimulating hormone (FSH) is synthesized and secreted from the anterior pituitary gland and plays an essential role in gonadal function. This gonadotropin binds to its receptor, the FSHR, expressed in the granulosa cells of the developing follicles, as well as in the Sertoli cells of the seminiferous tubules of the testis (1-3). Like other glycoprotein hormone receptors (luteinizing hormone/choriogonadotropin hormone receptor [LHCGR] and thyroid-stimulating hormone receptor), the FSHR is a G protein-coupled receptor (GPCR) that belongs to the highly conserved Rhodopsin-like subfamily of the GPCR superfamily (4). One particular feature of glycoprotein hormone receptors is the presence of a large extracellular domain (ECD), which is the site that recognizes and binds with high affinity the corresponding glycoprotein hormone and that, in turn, triggers a series of conformational changes in the transmembrane domains (TMDs) of the receptor protein (5), leading to receptor activation. Activation of the FSHR promotes, in turn, the activation of a complex network of intracellular signaling pathways regulated by several heterotrimeric G proteins (mainly the Gs protein) and other receptor-interacting proteins associated with the receptor (6).

Premature ovarian insufficiency (POI) is a heterogeneous disorder with an overall prevalence ranging from 1% to 3.7% (7, 8). Loss-of-function pathogenic variants of the FSHR may affect follicle development to varying degrees, leading to POI (9). To date, ∼45 or so pathogenic variants in the *FSHR* have been reported, which lead to distinct reproductive disorders; of these, ∼38 occurred in women with ovarian dysgenesis and POI (10-28). In men, pathogenic FSHR variants cause alterations in the quality and quantity of sperm (29). Inactivating FSHR variants are located along the protein sequence of the receptor, including the ECD, the TMDs, and the extra- and intra-cellular loops (ECL and ICL, respectively). The functional defects of several mutant FSHRs have been analyzed by expressing the abnormal receptors mainly in heterologous cell lines (12, 24, 30, 11, 14, 19, 23, 31), showing that both the location of the mutation in the receptor protein and the particular amino acid substitution determine the degree of functional impairment of the mutant FSHR.

Among those functionally defective FSHR variants, at least 15 are misfolded proteins that lead to impaired trafficking to the cell surface plasma membrane (PM) as disclosed by reduced PM expression of the mutant receptor (11, 12, 14, 18, 32-34). In addition to imaging studies, misfolding and defective trafficking of mutant gonadotropin receptors have been detected through studies showing rescue of PM localization and/or function by physical approaches or pharmacological chaperones (or pharmacoperones) (25, 35-39), which are small molecules that may penetrate the cell and function as a molecular framework promoting folding and correct routing of misfolded mutants or wild type proteins (40).

In the present study, we analyzed the *in vitro* effects of a novel allosteric agonist of the FSHR, CAN1405 [5-(9-(tert-butyl)-3-methoxy-2-(pyridin-3-yl)-5,6,8,9,10,11,12,13-octahydroazocino[4′,3′:4,5]pyrrolo[2,1-a]isoquinolin-14-yl)thiazole)] (Canwell Pharma Inc. USA) on PM expression and intracellular signaling of 13 FSHR pathogenic variants (A189V, N191I, D224V, D408Y, A419T, I423T, A462P, P504S, P519T, A575V, P587H, F591I, and L597I) (Fig. 1), leading to POI and expressed in human embryonic kidney 293 cells (HEK-293). We also characterized in detail the interaction of CAN1405 with the wild-type (WT) FSHR and 2 inactivating variants (D408Y and I423T), applying *in silico* studies, which represent an ideal strategy to characterize the conformational dynamics of glycoprotein hormone receptors occurring during binding to agonists and antagonists (41-43).

**Figure 1:**
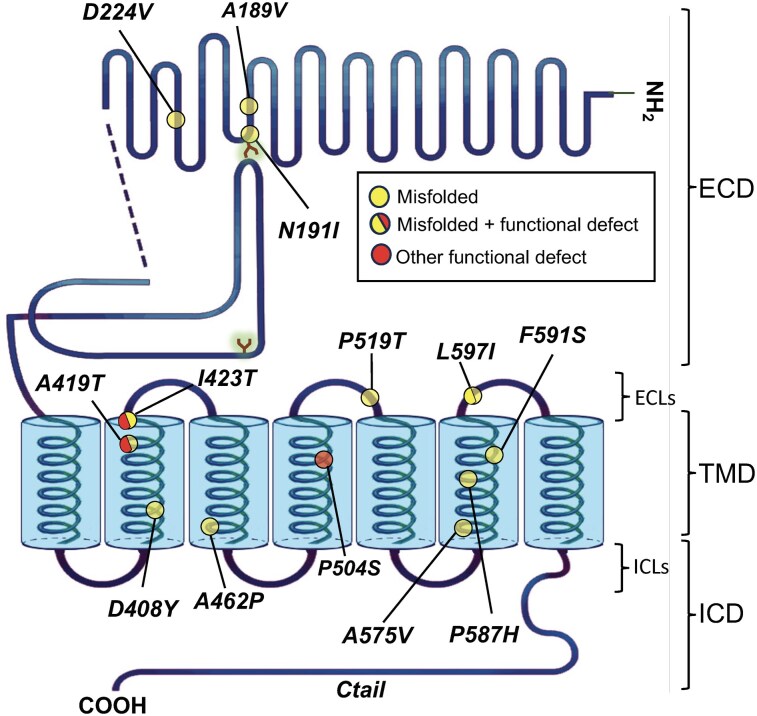
Schematic representation of the human FSHR showing the loss-of-function mutations analyzed in the present study (colored circles). Circles in yellow represent mutations that lead to misfolding and intracellular trapping of the mutated receptor as disclosed by biochemical and/or imaging studies showing decreased PM expression or intracellular accumulation of the receptor protein; yellow/red circles are residues in which both misfolding/intracellular retention and other functional defect have been demonstrated (13, 25); the red circle represents a pathogenic FSHR variant in which a biochemical defect has not been yet characterized, but that probably is a misfolded protein as suggested by *in silico* studies (22). Abbreviations: ECD, extracellular domain; TMD, 7 transmembrane domain; ICD, intracellular domains; ECLs, extracellular loops; ICLs, intracellular loops.

## Material and methods

### Construction of FSHR variants

Construction of the variants and the WT FSHR (bearing Thr at residue 307 and Asn at residue 680) was performed using the full-length WT human FSHR cDNA (GenBank Accession Number S59900) cloned into the mammalian expression vector pSG-5 (Invitrogen, Waltham, MA, USA). Residues at the positions of each FSHR variant were individually replaced with the mutant amino acids (A189V, N191I, and D224V in the ECD; D408Y, A419T, and I423T in the TMD2; A462P in TMD3; P504S in TMD4; A575V, P587H, and F591S in TMD6; and P519T and L597I in ECLs 2 and 3, respectively) (Fig. 1) by site directed mutagenesis. Pathogenic FSHR and WT variants cloned into the pSG5 vector (Agilent, Santa Clara, CA) were transiently expressed in authenticated HEK-293 cells by liposome-mediated endocytosis as previously described (44). Forward and reverse mutagenic oligonucleotide primers (Eurofins, Ebersberg, Germany) were designed following the cDNA sequence reported for the testicular WT FSHR (45) (Table S1 in Supplementary material (46)). The identity of all cDNA constructs and the correctness of the PCR products were checked by sequencing on a 3500 × L automated genetic analyzer (Applied Biosystems, Waltham, MA, USA). For transfection, large-scale plasmid DNAs were prepared using an Endofree maxiprep kit (Qiagen, Mexico City, Mexico).

### Cell culture and transfection of FSHR variants

HEK-293 cells were cultured in 60 mm dishes (Corning, Corning, NY) containing high-glucose Dulbecco´s modified Eagle´s medium (DMEM; Life Technologies, Inc., Grand Island, NY) supplemented with 10% fetal calf serum (FCS) (Invitrogen) and antibiotic (penicillin plus streptomycin) reagent (Life Technologies). Transient transfection with the WT FSHR, WT LHCGR (cloned in pcDNA3.1 expression vector (47)) or FSHR mutant cDNAs was performed by liposome-mediated endocytosis, as previously described (44). Twenty-four hours after transfection, HEK-293 cells were washed, counted, and replated in poly-d-lysine-coated (0.5 mg/well; Life Technologies) 24-well or 12-well dishes at a density of 75 000 or 200 000 cells/well, respectively, in supplemented DMEM and incubated for 24 hours in a humidified atmosphere of 5% CO_2_ at 37 °C before exposure to CAN1405, human recombinant FSH (recFSH) (Gonal F, Merck-Serono, Mexico City, Mexico) or highly purified human pituitary LH (NIDDK-hLH-I-SIAFP-1, National Hormone and Pituitary Program (Rockville, MD).

### Exposure of HEK-293 cells to CAN1405

Forty-eight hours after transfection, HEK-293 cells were exposed to CAN1405 (1 × 10^−6^ or 1 × 10^−7^ M, for western blotting or signaling assays, respectively) or vehicle (DMSO) dissolved in DMEM-HEPES 10 mM medium (Life Technologies) supplemented with antibiotics and 0.1% BSA for 24 hours in a humidified atmosphere of 5% CO_2_ at 37 °C. Concentrations of CAN1405 varied, depending on the particular experiment (see below) and on previous dose-response curves in HEK-293 cells expressing the WT FSHR. After the incubation period, cells were washed thrice with DMEM-HEPES 10 mM medium (Life Technologies) supplemented with antibiotics and 0.1% bovine serum albumin (BSA) (Sigma Aldrich, Mexico City, Mexico) and then processed for immunoblotting or exposed to recFSH.

### Western blots of the FSHR

Western blots of the FSHR were performed in HEK-293 cells transiently expressing WT or mutant FSHRs (cultured in 60 mm-well dishes) and pre-exposed to 10^−6^ M CAN1405 for 24 hours, as previously described (25). Cell lysates were electrophoresed in 7.5% sodium dodecyl sulfate polyacrylamide gels (SDS-PAGE), and western blotting of FSHRs was performed employing the highly specific anti-human FSHR antibody mAb106.105 at a 1:3000 dilution (48) (RRID: AB_2814841, http://antibodyregistry.org/AB_2814841) as primary antibody and anti-mouse IgG horseradish peroxidase conjugate (Jackson ImmunoResearch Laboratories Inc, West Grove, PA) (RRID: AB_10015289, http://antibodyregistry.org/AB_10015289) (1:10 000 dilution) as secondary antibody. Signal development was performed using the Clarity Western ECL substrate kit (Bio-Rad, Hercules, CA). Protein gel loading was visualized in a 1:10 000 mouse anti-glyceraldehyde-3-phosphate dehydrogenase (GAPDH) antibody (Merck Millipore, Burlington, MA) (RRID: AB_2107426, http://antibodyregistry.org/AB_2107426) and 1:15 000 goat-anti-mouse IgG conjugated with horseradish peroxidase (Jackson ImmunoResearch) (RRID: AB_10015289, http://antibodyregistry.org/AB_10015289) reprobed membrane.

### Reporter gene assay

For the reporter gene assay, HEK-293 cells were transiently co-transfected with the WT FSHR, LHCGR, or mutant FSHRs and the cAMP-sensitive pSOMLuc reporter plasmid (cyclic AMP response element (CRE)-luciferase reporter system), employing a previously described procedure (49). After transfection, cells were incubated with 10^−7^ M CAN1405 as described above, washed, and then exposed to 50 ng/mL recFSH or increasing concentrations of recFSH or human pituitary LH for 6 hours. After the FSH-exposed period, cells were lysed, and the luciferase activity was measured using a luciferase assay system (Promega). The light produced was measured in a luminescence counter and expressed as a fold increase over basal.

### ERK1/2 phosphorylation

HEK-293 cells transiently transfected with the WT or mutant FSHR cDNAs were exposed to 10^−7^ M CAN1405 for 24 hours and thereafter replated at a density of 200 000 cells/500 μL in 12-well culture plates (Corning) and tested for recFSH-stimulated ERK1/2 phosphorylation following previously described procedures (49-51). Briefly, after CAN1405 washing with serum-free medium, recFSH was added to cells and incubated for 5 or 120 minutes. At the end of the incubation period, cells were lysed in 2× Laemmli buffer and analyzed by western blot. The membranes were incubated overnight at 4 °C with rabbit anti-human phospho-ERK1/2 antibody (1:3000) (Cell Signaling Technology, Danvers, MA) (RRID: AB_331646 hsorA, http://antibodyregistry.org/AB_331646) and then with secondary anti-rabbit IgG horseradish peroxidase conjugate (GE Healthcare Life Sciences, Marlborough, MA) (RRID: AB_772206 hwsorA, http://antibodyregistry.org/AB_772206). Equal protein loading was confirmed in a membrane reprobed with primary polyclonal antibody against total ERK1/2 (1:10 000) (Santa Cruz Biotechnology Inc., Santa Cruz, CA, USA) (RRID: AB_2141292 hwsorA, http://antibodyregistry.org/AB_2141292). Signal development was performed using the Clarity Western ECL substrate kit (Bio-Rad). Results are expressed as the pERK/total ERK ratio calculated by densitometry of the blots employing the Chemidoc MP detection system (Bio-Rad).

The Stratagene PathDetect Elk1 trans-reporting system (Agilent Technologies, Santa Clara, CA) (Cat. 219005) was used following the manufacturer´s instructions. This system detects stimulation of Elk1 through phosphorylation of ERK1/2 after exposure to recFSH (50 ng/mL) or CAN1405 (1 × 10^−7^ M), and employs the pFR-Luc reporter plasmid to measure activation of the transcriptional activator in response to recFSH- or CAN1405-stimulated ERK1/2 phosphorylation. In this assay, cells were incubated for 4 hours at 37 °C, and included as a positive control 50 ng/mL of epidermal growth factor (EGF) (Sigma), which is a strong stimulator of MAPK-ERK phosphorylation (52).

### *In silico* studies

#### FSHR model

The initial coordinates of the FSHR were taken from the cryo-EM structure PDB:8I2G (43), in which the chain R corresponds to the receptor. The missing segment of the hinge region (HR) I279-M311 was included from a previous model (53). Palmitoyl tails were added to C644 and C646 (44), as well as disulfide bonds between the cysteine side chains of C18-C25, C23-C32, C275-C346, C276-C356, C442-C517, and C229-C338. Amino acid protonation states were set at pH 7.0. The full FSHR structure was inserted in a previously equilibrated bilayer patch of 1-stearoyl-2-docosahexaenoyl-sn-glycero-3-phosphocholine (SDPC) (54). Solvent water molecules and sodium ions for charge neutrality were added into a box of 120 × 120 × 160 Å^3^ in the *x, y,* and *z* directions, respectively. The FSHR in its apo (unbound) state contained ∼190 000 atoms. Two mutants that responded to CAN1405 exposure by increasing their PM expression (I423T and D408Y) were prepared from the FSHR native structure by replacing the side chain atoms of isoleucine with threonine and aspartate with tyrosine, respectively. We also prepared systems for the FSHR + CAN1405 complexes (holo [bound]-state). Because the crystal structure of the synthetic agonist was unknown, we devised a modeling strategy whereby the agonist could be included in the molecular dynamics simulation (MDS) protocol. We aimed to perform MDS to detect differences in the conformational dynamics due to the presence of the CAN1405 and whether the dynamics of the dysfunctional mutants could be modified when this synthetic agonist is located in the allosteric binding pocket.

#### CAN1405 model

An initial structure of the CAN1405 molecule was obtained with the Avogadro software 1.95 (55). The geometry was optimized using GAUSSIAN16 with the MP2 method and 6-31G(d,p) basis set representing the atomic orbitals (56). The atom charges were defined from the fit to the electrostatic potential (EP) surface of the optimized geometry. To verify the correspondence of the CAN1405 model with the synthetic agonist, we compared the experimental infrared spectra of CAN1405 against a density functional theory (DFT) calculation using the B3LYP functional along with a split valence polarized triple zeta (def2-TZVP (57)) basis set (58). The CAN1405 atom types compatible with the CHARMM36 force field (59) were generated by the SwissParam 2023 tool. Atom distances, angles, and dihedrals were inspected to be consistent with those of the optimized geometry. Force constants and non-bonding interaction parameters (eg, Lennard-Jones) were not further corrected at this stage of the MDS setup. To determine the initial coordinates of the CAN1405 in the FSHR allosteric binding site, we performed binding energy calculations using the GOLD software (60), with a 10 Å cavity around the side chain atoms of T449 located at the upper half of TMD3. This is a well-known binding site in family A of GPCRs and a typical target in drug design (61).

#### Molecular dynamics simulations

NAMD 3.0 software was used to generate the simulation trajectories in the isothermal-isobaric ensemble (NPT) (62). The Langevin dynamics was set to maintain a constant temperature of 300 K (63), and the Nosé-Hoover Langevin piston to maintain a constant pressure of 1 bar (64). Simulation box fluctuations were allowed independently in the x, y, and z directions (65). Non-bonding interactions were calculated with a cutoff of 12 Å, and a shifting function starting at 10.0 Å. A multiple time step integration for solving the motion equations was used, with 1 step for bonding interaction and short-range nonbonding interactions, and 2 steps for electrostatic forces, with a 2 fs time step. All hydrogen atoms were fixed using the SHAKE and RATTLE algorithms (66, 67). Electrostatic interactions were calculated with the PME method with a 4th order interpolation scheme on a ∼1 Å grid, and a 10^−6^ tolerance for the real contribution of the Ewald sum (68). The CHARMM36 force field parameters were set for the protein (69), and lipid atoms (70). The TIP3P potential was used to model the water molecules (71). Visualization of trajectories and analysis scripts were generated with the VMD software (72).

### Statistical analysis

Unpaired t-tests were applied to determine differences in mature to immature FSHR ratios before and after exposure to CAN1405, as well as between areas under the pERK/total ERK dose-response curves. Differences among changes in fold induction in the Elk-1 trans-reporter system in response to recFSH and CAN1405, as well as among recFSH-stimulated luciferase activity of each FSHR variant in the pSOMluc assay, were determined by one-way ANOVA followed by 2-sided pairwise t-tests. Within each FSHR variant, *P*-values from the 4 pairwise comparisons performed in the pSOMLuc assay were adjusted for multiple testing using Holm's method (73). Values of *P* ≤ .05 were considered statistically significant.

## Results

### Changes in FSHR PM expression in response to CAN1405

**Figure 2:**
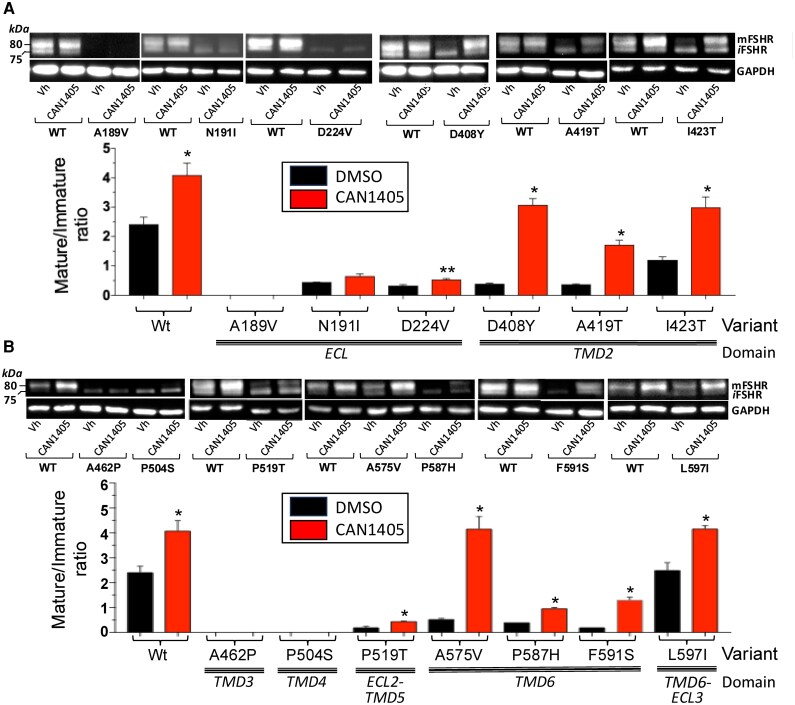
Mature to immature ratio of WT and FSHR mutants exposed to vehicle (DMSO) or CAN1405 as disclosed by WB. A. WT, A189V, N191I, D224V, D408Y, A419T, and I423T FSHRs. B. A462P, P504S, P519T, A575V, P587H, F591S, and L597I FSHRs. Each bar represents the mean ± SEM from 3 independent experiments in which each data point was performed in triplicate. *P < .01; **P < .05. Representative WBs of WT and each FSHR variant are shown on the top of the graphs.

Western blotting was performed to visualize changes in the relative PM expression of WT and mutant FSHRs as a result of exposure to CAN1405. The presence of 2 bands detected by the Mab106.105 specific anti-FSHR antibody, one that corresponds to the mature, membrane-expressed monomeric form of the receptor (M_r_ ∼80 kDa) and a second band that represents the immature, intracellular form of the FSHR (Mr ≤75 kDa) (74) were employed to calculate the mature/immature (M/I) FSHR ratio used as an index of changes in PM expression in response to the pharmacoperone (25). Western blot and densitometric analyses revealed that all FSHRs tested showed the band corresponding to the immature, mannose-enriched form of the receptor. Nevertheless, the expression levels of the mature and immature forms of the FSHR in response to CAN1405 exposure changed, depending on the particular location of the mutation across the receptor protein (Fig. 2A and 2B). Mutations in the ECD either did not respond (A189V) or responded marginally (as in the case of the N191I and D224V FSHR variants) to CAN1405 exposure by increasing the mature form of the FSHR. In the case of the FSHR A189V, neither mature nor immature forms of the receptor were detected by immunoblotting. In contrast, FSHRs bearing mutations in TMD2 (D408Y, A419T, and I423T), TMD6 (A575V), and at the boundary of TMD6 and ECL3 (L597I) showed a robust increase in the mature form of the receptor as a result of CAN1405 exposure. FSHRs with mutations at the TMD3 (A462P) and TMD4 (P504S) showed only the immature form of the receptor, whereas those located at the ECL2-TMD5 boundary (P519T) and TMD6 (P587H and F591S) showed a modest, albeit significant increase in M/I ratio. It is interesting to note that those FSH variants with amino acid substitutions involving a proline were recalcitrant or showed a very low response to the rescuing effect of CAN1405. In fact, in P504S, P519T, and P587H FSHR variants, the replacing residues were all disfavored substitutions for proline as it also was the case of the F591S variant. As shown in Fig. 2A and 2B, cells expressing the WT FSHR and exposed to CAN1405, exhibited a 1.6-fold increase (*P* < .01 vs vehicle-treated cells) in M/I FSHR ratio.

### FSH-induced pSOMLuc activation after CAN1405 pre-exposure

**Figure 3:**
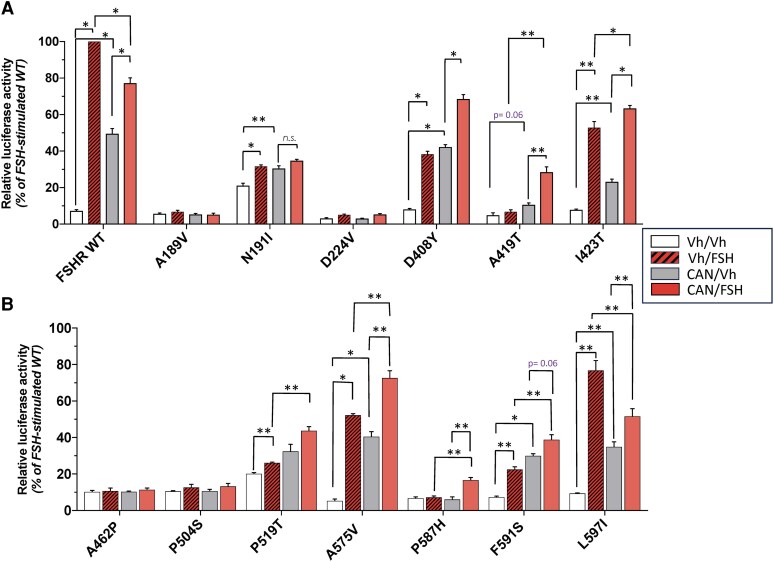
CRE-luciferase reporter activity in cells expressing the WT FSHR and 13 pathogenic FSHR variants after preincubation with 1 × 10^−7^ M CAN1405 or vehicle for 24 hours and then to 50 ng/mL recFSH or vehicle for 6 additional hours. A. WT, A189V, N191I, D224V, D408Y, A419T, and I423T FSHRs. B. A462P, P504S, P519T, A575V, P587H, F591S, and L597I FSHRs. *Vh/Vh:* Vehicle pre-exposed cells and then incubated with DMSO alone; *Vh/FSH:* Vehicle pre-exposed cells and then stimulated with recFSH; *CAN/Vh:* CAN1405 pre-exposed cells and thereafter incubated with DMSO (*ie,* basal relative luciferase activity after pre-exposure to CAN1405); *CAN/FSH:* CAN1405 pre-exposed cells stimulated with recFSH (*ie*, recFSH-stimulated luciferase activity after pre-exposure to CAN1405). Values are means ± SEM of 3 independent experiments in triplicate incubations. *P < .01; **P ≤ .05.

Exposure to CAN1405 for 24 hours and thereafter to 50 ng/mL of recFSH increased basal and/or agonist-stimulated luciferase activity in most of those FSHR variants whose PM expression rose as a result of CAN1405 exposure (Fig. 3A and 3B), and the increment in bioluminiscence correlated with their change in PM expression albeit with some differences due to different sensitivity of the experimental procedures. As expected, in those variants whose PM expression remained absent in response to CAN1405 exposure (A189V, A462P, and P504S in Fig. 2A and 2B), no significant changes in basal and/or FSH-stimulated luciferase activity were detected, whereas in those that showed a robust increase in PM expression as a result of CAN1405 pre-exposure (D408Y, I423T, A575V, and L597I FSHR variants), a prominent increase in luciferase activity basally (*ie,* in the presence of vehicle; CAN/Vh in Fig. 3) and in response to recFSH (CAN/FSH) was observed. In the case of the N191I and P519T variants, which showed a marginal (albeit significant in the latter) increase in PM expression upon CAN1405 exposure, changes in luciferase activity in basal conditions (N191I variant) or after exposure to CAN1405 and then to recFSH (P519T variant) were clearly detectable. Interestingly, the responses of the WT and the L597I variants to FSH stimulation, either in vehicle or CAN1405 pre-exposed cells, were very similar; both variants responded with a prominent increase in basal luciferase activity after CAN1405 exposure but exhibited a lower FSH-stimulated activity compared with cells not pre-exposed to the allosteric agonist. This latter finding may be due to increased internalization of the receptor provoked by FSH stimulation in the face of FSHR overexpression.

### FSH-stimulated ERK phosphorylation after CAN1405 pre-exposure

**Figure 4:**
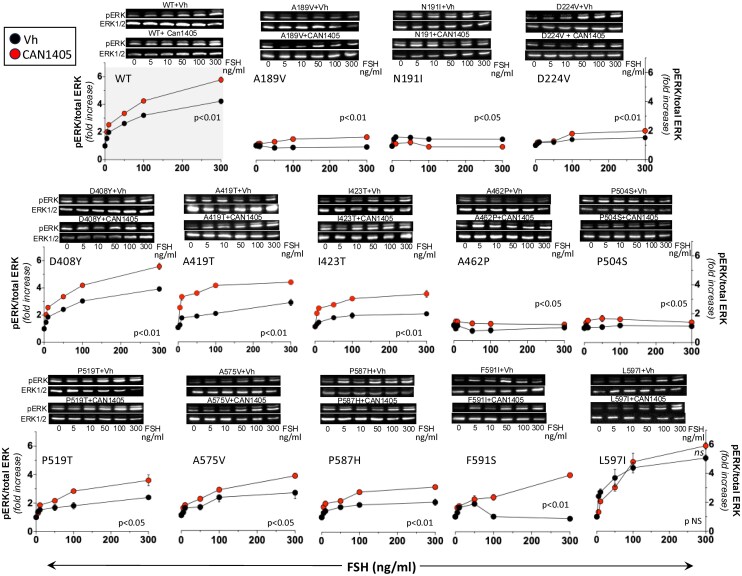
ERK1/2 phosphorylation in HEK-293 cells transfected with the WT FSHR or FSHR pathogenic variants and exposed to increasing doses of recFSH for 5 minutes after pre-exposure to CAN1405 (red circles) or vehicle (DMSO; black circles). Representative WBs of ERK phosphorylation stimulated by each FSHR are shown at the top of each graphic. Values are means ± SEM of 3 independent experiments in triplicate incubations. *P*-values for comparisons between the areas under the pERK/total ERK curves in vehicle vs CAN1405 pre-exposed cells are shown within the corresponding graph.

As shown in Fig. 4, the WT FSHR, as well as all but the N191I and L597I variants, increased ERK1/2 phosphorylation as disclosed by changes in the area under the pERK/total ERK curve (AUC) after pre-exposure to CAN1405 for 24 hours and then to 0 to 300 ng/mL recFSH during 5 minutes. The magnitude of the changes detected, however, varied depending on the location of the mutation and the nature of the amino acid substitution. The increase in AUC of ERK1/2 phosphorylation was marginal (albeit statistically significant) in the A189V, D224V, A462P, and P504S variants, which contrasted with their complete absence of response in FSH-stimulated luciferase activity in the pSOMLuc reporter assay. This finding may be due to differences in both sensitivity and doses of recFSH employed in the 2 assays. Compared to CAN1405 unexposed cells, the remaining FSHR variants (WT, D408Y, A419T, I423T, P519T, A575V, P587H, and F591S) pre-exposed to CAN1405 and then to recFSH, exhibited a clearly detectable dose-dependent increase in pERK during recFSH exposure, whereas in the case of the L597I variant, the increase was marginal and not significant (Fig. 4, lower panel).

### Effect of CAN1405 stimulation on the FSH and LHCG WT receptors

**Figure 5:**
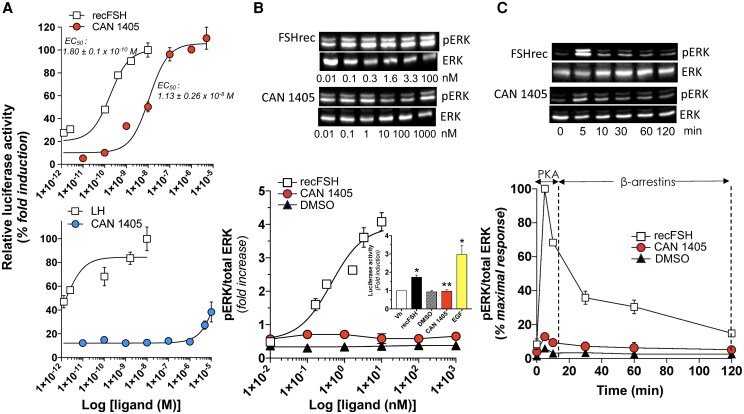
Relative luciferase activity (pSOMLuc) in HEK-293 cells transfected with the WT FSHR or LHCGR cDNA and exposed to increasing doses of recFSH or CAN1405 (a, upper panel) or human pituitary LH (a, lower panel). B: ERK1/2 phosphorylation in HEK-293 cells transfected with the WT FSHR cDNA and exposed for 5 minutes to increasing doses of recFSH, CAN1405, or vehicle (DMSO). *Inset:* Luciferase activity in the luciferase-based Gal-Elk reporter system employed to monitor activation of Elk1 by pERK1/2 (*P < .01 vs DMSO and CAN1405; **P NS vs DMSO). *Top:* Representative WB of ERK1/2 phosphorylation after exposure to recFSH or CAN1405. C: Time-course activation of ERK1/2 in cells expressing the FSHR and stimulated during 120 minutes with recFSH (50 ng/mL [1.6 nM]), CAN1405 (1 × 10^−7^ M), or vehicle. Note the marginal increase in pERK/total ERK ratio during the first 15 minutes of exposure to CAN1405 and the virtual absence of response during the ensuing 105 minutes of incubation. *Top:* Representative WB of ER1/2 phosphorylation in response to fixed doses of recFSH or CAN1405 during 120 minutes. Data on graphs are means ± SEM of 3 independent experiments in triplicate incubations.

Exposure of HEK-293 cells to increasing concentrations of recFSH or CAN1405 resulted in a dose-dependent increase in cAMP-provoked pSOMLuc expression, with EC_50s_ of 0.18 ± 0.02 nM and 11.3 ± 3.23 nM (mean ± SD), respectively (Fig. 5A). The effect of the allosteric agonist to stimulate cAMP-provoked pSOMLuc expression was also analyzed in HEK-293 cells transiently expressing the LHCGR. Incubation of HEK-293 cells expressing the LHCGR with increasing concentrations of pituitary LH (1-300 ng/mL) elicited a robust dose-dependent increase in luciferase activity (Fig. 5A lower panel). By contrast, exposure of cells to increasing amounts of CAN1405 resulted in partial pSOMLuc expression (up to ∼40% of that achieved with the maximal dose of LH) when stimulated with the highest CAN1405 dose (10 *μ*M), thus behaving as a weak allosteric agonist for this receptor.

To analyze the response of ERK1/2 MAPK phosphorylation (which is stimulated by FSH through activation of both cAMP/PKA and β-arrestin 1/2 signaling pathways) to recFSH or CAN1405 stimulation, HEK-293 cells expressing the WT FSHR were exposed to increasing doses of recFSH (0-300 ng/mL [0-10 nM]) or CAN1405 (0-500 ng/mL [0-10^−6^ M]) during 5 minutes (Fig. 5B) or to 50 ng/mL (1.6 nM) recFSH or 10^−7^ M CAN1405 for 0 to 120 minutes (Fig. 5C). As shown, stimulation with recFSH provoked a robust dose-dependent increase in ERK1/2 phosphorylation, whereas exposure to CAN1405 provoked a marginal, non-significant change in this particular readout. Furthermore, luciferase activity in the luciferase-based Gal-Elk reporter system employed to monitor activation of Elk1 by pERK1/2, remained unchanged after 4-hour exposure to 10^−7^ M CAN1405 at 37 °C. In contrast, stimulation with epidermal growth factor (employed as a positive control) or 50 ng/mL recFSH resulted in a significant increase in luciferase activity whose promoter harbors the Elk1 activation site (Fig. 5B, *inset*). In the time-course experiment of ERK1/2 phosphorylation stimulated by exposure to fix amounts of the FSHR agonists, the WT FSHR exhibited a biphasic response, that is, a short-lasting response (5-15 minutes) (mainly, but not exclusively mediated by the cAMP-PKA pathway), followed by a late response (15-120 minutes), which is primarily dependent on activation of β-arrestin 1/2 signaling cascade (Fig. 5C). In contrast, stimulation with CAN1405 provoked only a marginal increase in ERK1/2 phosphorylation during the early phase, but no increase in the late, β-arrestin 1/2-dependent phase. Taken together, these results indicated that CAN1405 behaved as a biased agonist toward the cAMP-regulated signaling pathway in HEK-293 cells transiently expressing the FSHR.

### In silico studies

#### Structure and docking of CAN1405 in the WT FSHR

We first calculated the infrared (IR) spectra of CAN1405 by the calculation of vibrational frequencies [Fig. S1 (46)]. From the comparison of the IR spectra and the calculated frequencies, it was possible to identify signatures of vibrations at 3000 cm^−1^, 1600 cm^−1^, 1300 cm^−1^, and 850 cm^−1^ in both spectra. From the IR calculation, we concluded that the CAN1405 structure was a reasonable approximation for the geometry and charge distribution used in the docking and MDS computational protocols.

**Figure 6:**
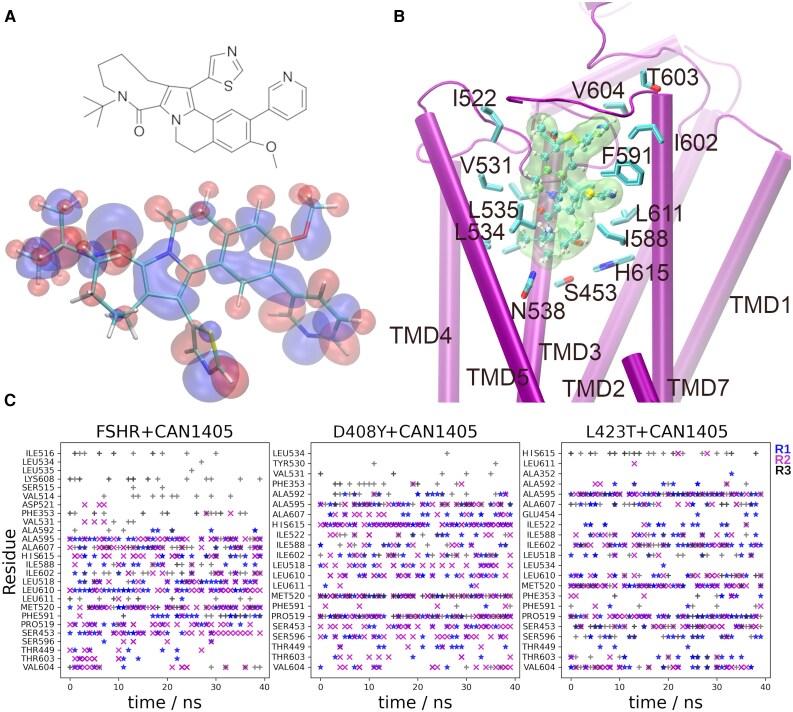
A. Optimized structure of the CAN1405 and its (SMILES) formula. Systematic name of CAN1405 is 4-*tert*-Butyl-15-methoxy-14-(3-pyridyl)-10-(1,3-thiazol-5-yl)-1,4-diazatetracyclo [9.8.0.0^2,9^.0^12,17^] nonadeca-2(9),10,12,14,16-pentaen-3-one. Isosurfaces for the electrostatic potential are shown in blue (−50 kT/e) and red (+50 kT/e). B. Position of CAN1405 (green surface and ball-sticks; color code C-cyan, H-white, N-blue; S-yellow) in the allosteric binding site of FSHR (purple cartoons). Side chain of residues in the binding pocket at 3.5 of CAN1405 are shown (licorice; color code C-cyan, N-blue; S-yellow). C. Contact maps for CAN1405 at the allosteric site of the WT FSHR and mutants D408Y and I423T, in the last 40 ns of trajectories R1-R3. Main interactions include mainly residues in TMD3 (S453, G454, T449), TMD6 (F591, A592, A595, S596, I588), and TMD7 (I602, V604, A607, L610, H615), as well as few residues in the NH_2_-Terminus (F353), TMD5 (G353), and ECL2 (M520, I522).

Figure 6A shows the structural formula of CAN1405, its optimized geometry, and the EP isosurfaces with positive and negative potentials. As expected, negative regions located at electronegative atoms (oxygen and nitrogen) and positive regions were located mainly at hydrogen atoms. Carbon atoms bear positive or negative charges according to bonding partners, whereas sulfur bears a slight negative charge. From the optimized geometry of CAN1405, the docking calculation allowed us to determine the best pose for the agonist in the allosteric site of the FSHR (Fig. 6B). Side chain interactions of the WT, D408Y and I423T FSHRs with CAN1405 were identified as function of the simulation time for the last 40 ns of the trajectories R1-R3 (Fig. 6C). Docking studies of CAN1405 in the FSHR showed that this allosteric agonist mainly interacts with residues located in the upper third of TMDs 3, 6, and 7 (Fig. 6B), including residues S453, G454, and T449 in TMD3; F591, A592, A595, S596, and I588 in TMD6; and I602, V604 T604, A607, L610, and H615 in TMD7. Few residues were identified in the NH_2_-terminus (F353), TMD5 (G353), and ECL2 (M520 and I522) (Fig. 6C). Although most of the amino acid contacts of CAN1405 in the WT FSHR were conserved in the D408Y and I423T FSHR variants, interaction of CAN1405 with H615 was detected in the I423T receptor but only in R3 (Fig. 6C, right panel). This decreased interaction is highlighted, given that H615 is an important residue involved in selectivity and interactions with allosteric agonists (43).

#### Conformational changes in the WT, D408Y, and I423T FSHRs induced by CAN1405

**Figure 7:**
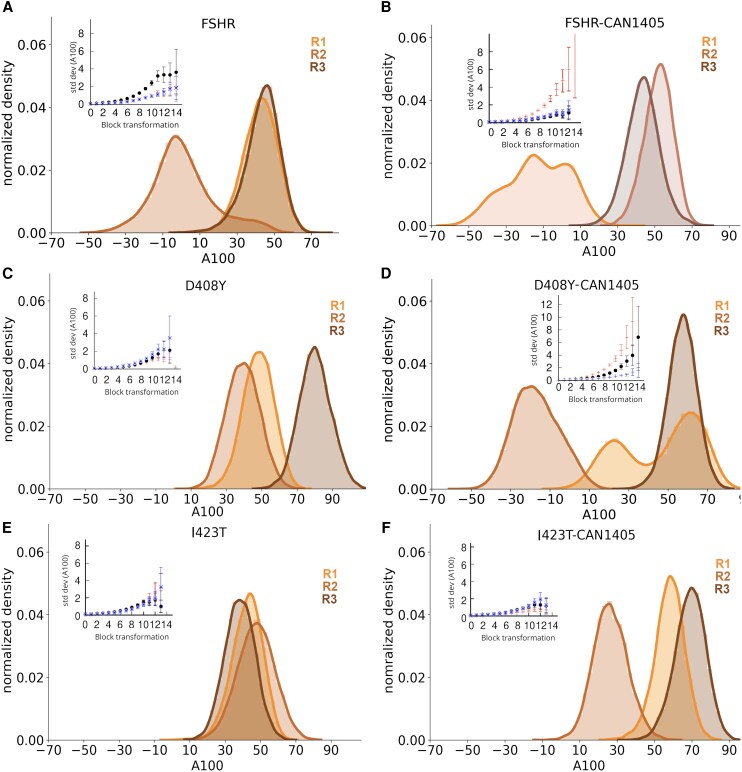
Distribution of the A100 index for 3 MD replicates (R1-3) of the WT FSHR (A), WT FSHR + CAN1405 (B), D408Y mutant (C), D408Y mutant+CAN1405 (D), I423T mutant (E), and I423T mutant+CAN1405 (F). Insets show the standard deviation calculation using the blocking average method. Most of the standard deviations showed a constant value after 11 block transformations. From the A100 index the conformational states for the active (A < 55), partial active (0 < A100 < 55), and inactive states (A100 < 0) were identified. The WT FSHR showed a flexible structure, while the mutants were rather rigid. CAN1405 induced an enhanced dynamics as the receptors populated alternative states from those of the apo state.

The A100 index allowed us to classify the receptor conformations generated in 3 independent replicates at the ∼4 × 10^2^ ns time scale. Values lower than zero, *ie,* A100 < 0, correspond to the inactive state, whereas values A100 > 55 correspond to the active state, and within 0 < A100 < 55 to the partially activated state (75). From distributions of the A100 index in trajectories R1-R3 of the WT FSHR and WT FSHR + CAN1405 complex (Fig. 7A), it appears that the conformations of the TM domains populated active, inactive, and partially active states. The active state was well preserved, given the maxima of the distributions of R1 and R3 in WT FSHR, and R2 and R3 in the presence of CAN1405 (Fig. 7A and 7B); nonetheless, partially activated states were also detected. According to the A100 index, the receptor dynamics in the membrane environment showed fluctuations sufficent to overcome the energy barrier for the transition of active to inactive in R2 of the WT FSHR and in R1 of the WT FSHR + CAN1405 complex (Fig. 7B). This observation is interesting because we never included any potential bias to induce such a transition. To further determine whether the A100 index reached a representative average, we used the blocking average method (76) to measure the standard deviation and its error. For a set of independent values, the standard deviation reaches a constant value (see insets in Fig. 7). The broader distribution of R2 in the WT FSHR was consistent with the larger standard deviation relative to R1 and R3. In R1 of WT FSHR + CAN1405, the standard deviation reached a constant value within error bars, showing a broader distribution relative to R2 and R3. In the presence of CAN1405, the conformational dynamics of the WT receptor was preserved in most of the trajectories. Interestingly, in system D408Y + CAN1405 (Fig. 7D), the receptor changed its conformation to partially activated (R1) or inactive states (R2), featuring distinguishable conformations. In R1 of WT FSHR + CAN1405, the distribution was broad, with 2 maxima and a shoulder, suggesting alternative inactive states not accessible in the unbound WT FSHR. It is possible that the presence of CAN1405 may enhance the transitions to different states in comparison to the apo state, as suggested by the broader and multimodal distributions or shifts of the distribution's peaks. In fact, relative to the I423T variant, the distributions in the I423T + CAN1405 shifted to larger A100 values in R1 and R3, (41.3 vs 59.1 and 38.5 vs 69.4, respectively), while in R2 decreased (47.5 vs 27.2). The averages and standard deviations for all FSHR setups with and without CAN1405 are shown in Table S2 (46). From these observations, the presence of CAN1405 enhances the conformational dynamics of the TMD to the extent that mutants D408Y and I423T sampled regions of the conformational space apparently not accessible in the apo state.

Another characterization of the active to inactive transitions in the WT FSHR was based on the dynamics of the microswitches (77). For comparison, Table S3 (46) shows distances for the known structures of WT FSHR structures. Calculations of microswitch distances allowed us to identify rearrangements of the TMDs of the WT FSHR. Figure S2 (46) shows projections of the helix-helix distances whereby the dynamics of the TMDs were revealed in the apo and holo states. In these plots, a high density of points represents highly populated conformations or free energy wells, while sparse distributions represent low populated conformations or probably transition states at free energy barriers. Some trajectories showed dynamic distance distributions and shifts from distances of the cryo-EM structure (Table S3 (46)); for example, the distribution of the TMD6-TMD4 distance in R2 and R3 increased by ∼8 Å; TMD5-TMD7 in R1 and R3 increased by ∼6 Å; and TMD6-TMD3 in R2 and R3, by ∼5 Å. Overlap among distance distributions in TMD6-TMD3, TMD7-TMD4, and TMD6-TMD5, as in R2 and R3, represents conformations with similar distance features. In R1, some distances remained close to that of the initial cryoEM structure: TMD6-TMD5, TMD6-TMD3, TMD2-TMD7, and TMD7-TMD4. The most consistent displacement in all replicates involves a distance decrease between TMD6 and TMD3, accompanied by a slight distance increase between TMD6 and TMD5, probably linked to an inward motion of TMD6 in transition toward the inactive state. When CAN1405 was in the binding pocket, the TMD-TMD distance distributions showed similar features as the FSHR in the apo state, with shifted TMD-TMD distances relative to those of the cryo-EM structure and with significant overlap in the distance distributions. Both the apo and holo states of the FSHR preserved a highly dynamic distance distribution of the TMD regarding the identified as microswitches important for the receptor's active-inactive transition in family A of GPCRs.

TMD-TMD distance distributions in D408Y were relatively close to the cryo-EM structure (Fig. S3 (46)). The largest motion was a decrease of the TMD3-TMD6 distance, from 34 to 29 Å, with a slight increase of the TMD6-TMD5 up to 14 Å in R2. When CAN1405 was in the binding pocket, other conformations were accessible for the mutant. Distance TMD2-TMD7 increased from 9 to 14 Å, and that of TMD6-TMD4 from 30 to 38 Å. Interestingly, each distribution reached 1 main maximum representing a stable conformation in each case, with negligible overlap among the distributions. In the case of the I423T variant, TMD-TMD distance distributions preserved mainly the initial conformation, with slight shifts and significant overlaps [Fig. S4 (46)]. Conformations of the R2 showed differences in TMD6-TMD5, TMD3-TMD6, and TMD3-TMD6 relative to conformations of R1 and R3. When CAN1405 was present in the I423T binding pocket, the distributions showed similar trends as in the apo state, although with slightly increased dynamics. The overall view that emerges from the comparison of the apo and holo states of the FSHR and mutants can be summarized as follows: (1) the FSHR showed highly flexible TMDs with broad distance distributions and shifts of the TMD distances involving the activation microswitches; (2) mutations D408Y and I423T impact the TMD dynamics by preserving the initial active conformation, which is an evidence of an impaired conformational flexibility; and (3) the agonist CAN1405 preserved the TMD dynamics of the WT FSHR, while it slightly enhanced the dynamics of the mutant receptors by populating conformational states not accessible in the apo states. From these comparisons, the role of CAN1405 seems to be related to the enhancement of the conformational dynamics, which seems apparently dysfunctional in the case of the mutants D408Y and I423T.

## Discussion

It has been shown that mutations in GPCRs may compromise the synthesis of the receptor protein and the function of domains involved in agonist binding, receptor activation, or coupling to effectors. Mutations in these membrane receptors may also lead to misfolded, trafficking-defective proteins unable to transport from the endoplasmic reticulum (ER) to the PM (78). These functional defects are not mutually exclusive as 1 mutation may provoke functional defects in both intracellular traffic and any other function, depending on the specific location of the amino acid replacement. Several loss-of-function mutations in GPCRs involved in reproductive function have been reported, some of which lead to misfolded variants with a trafficking defect that can be corrected with small-molecule pharmacoperones (34-36, 38, 79-81).

Thirteen misfolded FSHR variants leading to POI were tested for PM expression and functional rescue by the small-molecule CAN1405. The 3 variants located in the ECD (A189V, N191I, and D224V; all within the leucine-rich repeat domain [LRR]) failed to traffic to the PM or showed a marginal increase in expression in response to CAN1405. This might be due to: *a.* the severe conformational alteration in the ECD provoked by the mutation; and/or *b.* failure of the allosteric agonist to stabilize a conformation compatible with ER export because its sites of interaction with the receptor are relatively distant from the region at which the mutations are located. In fact, the interaction sites of CAN1405 with the FSHR were mainly located in the upper third of the TMDs, and only very few interactions with the ECLs and ECD regions were identified. This is consistent with observations showing that the allosteric FSHR compound 21F binds to the top half of the TMD as revealed by the cryo-EM structure of FSHR (43). In the case of A189V and N191I variants, the replacing amino acid may compromise the integrity of the 189AFNGT193 sequence motif associated with the putative glycosylation site at N191. This posttranslational modification is apparently important for proper LRR domain formation at its α-helical portion, and thus mutations at or near this motif may potentially lead to decreased efficiency of receptor folding and increased degradation of the protein precursor, as demonstrated in rat luteinizing hormone receptors bearing mutations at putative glycosylation sites (82). Nevertheless, both the A189V and N191I FSHR variants are not completely devoid of signaling activity, as we found in the pSOMLuc activation and the ERK 1/2 phosphorylation assays. In fact, the A189V FSHR variant has been associated with preferential β-arrestin mediated signaling (49) when expressed at low levels, whereas the N191I variant may show substantially decreased (albeit still present) FSH-stimulated cAMP response (33, 83). The failure of the A189V variant to be rescued with CAN1405 contrasts with previous findings in which pre-treatment with an LHCGR allosteric agonist (Org 41841) modestly increased high-dose FSH-stimulated cAMP production by that mutant (84). In this regard, no evidence was shown that the allosteric agonist in fact increased cell-surface PM expression of the variant receptor. In agreement with our data, other laboratory manufactured mutations in the human FSHR ECD that also failed to be expressed on the cell surface were not rescued with Org 41841 (85, 86), nor PM expression of the same ECD variants tested in the present study could be rescued by the small molecule CAN1404, except probably the N191I which showed a marginal but still detectable response to this latter agonist (36).

The response to CAN1405 pre-treatment of variant FSHRs with mutations in the TMD was variable. Two variants located in TMD3 (A462P) and TMD4 (P504S) were either recalcitrant or marginally responsive to CAN1405 as assessed by PM expression or FSH-stimulated signaling after pretreatment with the allosteric agonist. Both variants involve a proline residue whose substitution or presence as a replacement residue may provoke a distorted conformation in the protein, given that this amino acid is connected to the backbone of the protein twice, thereby favoring the formation or disruption of tight turns in α-helices. Other variants involving a proline residue (P519T and P587H) were also poorly rescued by CAN1405; in fact, proline residues at these locations are key structural elements of the FSHR, and their substitutions may disrupt FSHR stability (43). Furthermore, in addition to their role as important structural features of the receptor, proline residues at these positions are also involved in the interaction of CAN1405 with the WT FSHR (Fig. 6C, left panel), and their substitution may thus alter this interaction. In contrast, PM expression and function of variants in TMD2 (D408Y and I423T) and TMD6 (A575V) were effectively rescued by the pharmacoperone CAN1405, except for the A419T FSHR variant, whose rescue was rather modest. Although in the original report (13), the PM expression of that mutant was similar to that of the WT receptor, more recently, it was found that its cell surface expression is, in fact, severely reduced (36), as confirmed in the present study. Furthermore, molecular modeling has revealed that the replacement of the hydrophobic alanine (which facilitates PM insertion of the receptor) with the polar threonine in position 419 provokes a significant conformational change (87), which might be responsible for the poor PM expression of the variant and the modest response to CAN1405. In this regard, our docking study did not show any interaction of CAN1405 with the WT FSHR at the A419 residue. Finally, although the PM expression of the FSHR F591S variant was only modestly rescued by pre-treatment with CAN1405, its FSH-stimulated signaling significantly improved as detected in both readouts employed.

It was interesting to find that the behavior of the L597I variant was very similar to that of the WT receptor. This variant was detected in a woman with POI (88), and *in vitro* studies in HEK293 cells found that PM expression, FSH-induced cAMP production, and ERK1/2 phosphorylation were significantly reduced in those cells expressing this mutant. The reason for this disagreement between both studies is not clear, and the possibility exists that a second mutation that may potentially affect folding, trafficking, and function of that FSHR variant was missed by the investigators of the original report (88). Another interesting observation was that pre-treatment of cells expressing the WT FSHR with CAN1405 significantly increased both PM expression and signaling of the WT receptor by 40% to 60%. This finding differs from those of a previous study employing the allosteric agonist CAN1404 (36) and could be due to differences between the interaction sites of the 2 agonists with the FSHR. Different interactions may lead to distinct conformational changes in the receptor molecule that may impact the response of the WT receptor to the rescuing effect of the pharmacoperones. Future docking studies focusing on differences in interaction sites between the FSHR and these 2 pharmacoperones are necessary to explain the different responses of the FSHR variants and the WT FSHR to these small molecules.

We also characterized the structural and functional features of CAN1405, a novel allosteric agonist of the human FSHR with pharmacoperone properties. The interaction of CAN1405 with the WT FSHR was studied through both biochemical and *in silico* approaches. The biochemical studies showed that CAN1405 stimulated a dose-dependent increase in pSOMLuc activation similar to that elicited by recFSH (albeit with a lower potency) without a clearly detectable effect to stimulate ERK1/2 phosphorylation. In this setting, CAN1405 behaved as a biased agonist toward the cAMP-PKA signaling pathway. This finding is similar to that exhibited by the allosteric agonist CAN1404, which provoked a very modest inositol phosphate accumulation response (Gα16-mediated) at the WT FSHR, despite a robust stimulation of cAMP-mediated signaling (36). The biased signaling exhibited by both CAN compounds, as well as their preferential interaction with the FSHR, offers a therapeutic opportunity to exogenously modulate the intensity of the FSH stimulus *via* fine-tuning regulation of receptor-associated signaling pathways.

In the present study, we implemented a set of strategies to detect the conformational dynamics of the WT FSHR with and without CAN1405, and also included the variants D408Y and I423T, which exhibited a positive response to the rescuing effect of CAN1405. From our modeling approach based on the optimum geometry and charge distribution using electronic structure calculations, we observed that the receptor-agonist complex structure was well preserved and stable during the simulation stage. This implementation allowed us to detect how the allosteric agonist CAN1405 modulates the receptor function when mutations at the second TMD provoke structural changes and impaired flexibility. The structural flexibility of these FSHRs is an important feature as they can bind to different intracellular effectors, respond to diverse ligands, and trigger different cellular signals, thereby displaying rich conformational dynamics. In fact, the WT FSHR was highly dynamic in both the apo and holo states. In contrast, we detected an enhancement of the dynamics when CAN1405 was present in the allosteric site of the D408Y and I423T FSHR mutants. Comparisons of the TMD-TMD microswitches and the A100 index suggest that the presence of CAN1405 enhanced the conformational dynamics by making accessible conformational states that were not populated in the rather rigid structure of the apo states. However, in the I423T variant, the increased dynamics were not enough to observe active-to-inactive transitions. In the D408Y-CAN1405 system, on the other hand, the induced dynamics of the pharmacoperone were more evident as the R2 run showed a full transition and R1 showed 2 maxima representing 2 stable conformations. In the WT FSHR, CAN1405 induced alternative conformations that could explain intracellular signal triggering when the synthetic ligand is used as a stimulus.

In summary, the allosteric agonist CAN1405 behaved as a pharmacoperone to rescue cell surface expression and function of upward trafficking-defective, misfolded human FSHRs leading to POI. In general, those variants with mutations in the TMDs were more amenable to rescue by CAN1405 than trafficking-defective receptors with mutations in the ECD. CAN1405 also increased plasma membrane expression and function of the WT FSHR, indicating that, normally, a portion of this receptor is incompletely folded and trapped inside the cell. Stabilization of an active receptor conformation compatible with endoplasmic reticulum export appears to underpin the rescue phenomenon of CAN1405, as shown by *in silico* analysis of WT and mutant FSHRs. The overall data represent a therapeutic opportunity for those patients bearing pathogenic variants of the FSHR as well as for women who exhibit a poor response to exogenous FSH stimulation in assisted reproduction programs.

## Data Availability

Datasets generated during and/or analyzed during the current study are not publicly available but are available from the corresponding author on reasonable request.
